# Druggable Targets in Cyclic Nucleotide Signaling Pathways in Apicomplexan Parasites and Kinetoplastids against Disabling Protozoan Diseases in Humans

**DOI:** 10.3390/ijms20010138

**Published:** 2019-01-02

**Authors:** Annette Kaiser

**Affiliations:** Medical Research Centre, University Duisburg-Essen, Hufelandstrasse 55, 45147 Essen, Germany; kaiser@microbiology-bonn.de; Tel.: +49-151-4121-9343

**Keywords:** small GTPases, cAMP, cGMP, GPCR, Apicomplexa, kinetoplastids, drug discovery

## Abstract

Cell signaling in eukaryotes is an evolutionarily conserved mechanism to respond and adapt to various environmental changes. In general, signal sensation is mediated by a receptor which transfers the signal to a cascade of effector proteins. The cyclic nucleotides 3′,5′-cyclic adenosine monophosphate (cAMP) and 3′,5′-cyclic guanosine monophosphate (cGMP) are intracellular messengers mediating an extracellular stimulus to cyclic nucleotide-dependent kinases driving a change in cell function. In apicomplexan parasites and kinetoplastids, which are responsible for a variety of neglected, tropical diseases, unique mechanisms of cyclic nucleotide signaling are currently identified. Collectively, cyclic nucleotides seem to be essential for parasitic proliferation and differentiation. However, there is no a genomic evidence for canonical G-proteins in these parasites while small GTPases and secondary effector proteins with structural differences to host orthologues occur. Database entries encoding G-protein-coupled receptors (GPCRs) are still without functional proof. Instead, signals from the parasite trigger GPCR-mediated signaling in the host during parasite invasion and egress. The role of cyclic nucleotide signaling in the absence of G-proteins and GPCRs, with a particular focus on small GTPases in pathogenesis, is reviewed here. Due to the absence of G-proteins, apicomplexan parasites and kinetoplastids may use small GTPases or their secondary effector proteins and host canonical G-proteins during infection. Thus, the feasibility of targeting cyclic nucleotide signaling pathways in these parasites, will be an enormous challenge for the identification of selective, pharmacological inhibitors since canonical host proteins also contribute to pathogenesis.

## 1. Introduction

Protozoan parasites can be subdivided into two major groups, i.e., the Excavates with flagellar structures and the group of Alveolates which contain membranous vesicles, so called alveoli, located beneath the plasma membrane. In the taxonomic rank the most important phylum with respect to disease in the group of Alveolates are the Apicomplexa comprising the genera *Plasmodium*, *Cryptosporidium* and *Toxoplasma*. In contrast, the major group of Excavates consists of the phylum Euglenozoa comprising the genera *Leishmania* and *Trypanosoma* known as the kinetoplastids with large, massed DNA called the kinetoplast.

The cAMP signaling pathway and multiple activated factors are involved in regulating numerous physiological processes, including growth, reproduction, differentiation and apoptosis. Disruption of this pathway can lead to treatment of the disease. The physiological functions depend on the targeted tissues, cells and organs. In mammals, for example, cAMP has multiple roles ranging from auditory function to mediating hormone action. cGMP is a central player in processes such as cardiac function and light detection in the eye. Various physiological functions are also attributed to G-proteins from knock-out studies in mice. G_s_ and G_i_ subunits contribute to cardiac functions such as contractility. Gq and G_12_ have multiple functions like cerebral development, cardiomyocyte formation, craniofacial development and parathyroidism. GPCRs are the most intensively studied drug targets due to their involvement in pathophysiological processes. 

**Figure 1:**
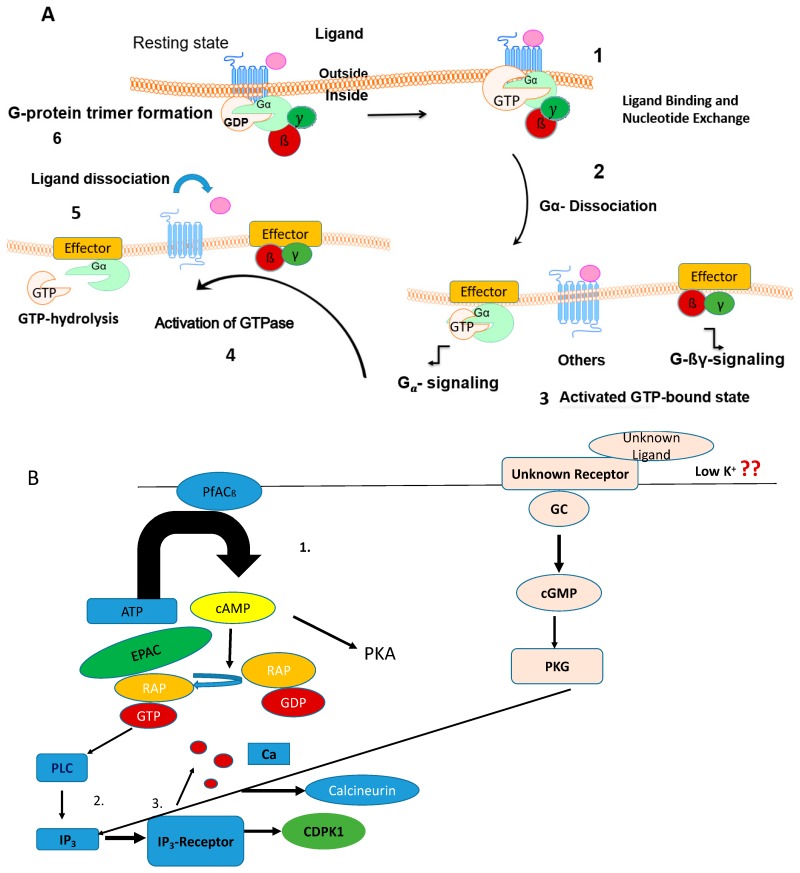
Comparison of a canonical, cAMP-signaling pathway in the human host cell and in Apicomplexan parasites: (**A**) Activation/reactivation cycle of a heterotrimeric G-protein in the context of G-protein-coupled receptor (GPCR) signaling: 1. binding of a ligand to the receptor causing a conformational change, 2. GDP bound to the alpha-subunit is exchanged to GTP, 3. dissociation of the alpha-subunit from the G_βγ_-dimer and the receptor, 4. Formation of a complex between the G-alpha subunit or the G_βγ_-dimer and the effector molecule 5. Activation of a GTPase that hydrolyzes GTP to GDP under the control of a regulator of G-protein signaling, 6. Trimer formation of the different G-protein subunits. (**B**) Non-canonical cyclic nucleotide signaling pathways in *Plasmodium*. The exchange protein (EPAC) pathway (left side of the figure): 1. during egress and invasion of merozoites PfACβ triggers cAMP levels fostering the EPAC pathway which in turn leads to phosphorylation of Rap-GDP to Rap-GTP. 2. Rap-GTP activates phospholipase C (PLC) to produce inositol triphosphte (IP_3_) that binds to the IP_3_ receptor. 3. Activation of the IP_3_ receptor leads to a release of calcium ions. Calcium ions bind to calcineurin and activate calcium-dependent protein kinase 1 (CDPK1). Middle part of the figure: besides the EPAC pathway cAMP activates protein kinase A. Right part of the figure: at low potassium concentrations an unknown ligand binds to the SR25 receptor which mediates the signal to a guanylyl cyclase that activates protein kinase G. Protein kinase G (PKG) acts in concert with a stage specific calcium dependent kinase.

Research on G-protein coupled receptor (GPCR)-mediated signaling in protozoan parasites has been intensified during recent years. One widely used principle of signal transduction in eukaryotes is the signaling through GPCRs [1]. G-proteins represent a heterogenous group of proteins. In canonical GPCR-coupled pathways, binding of a ligand (agonist) to a receptor leads to a conformational change in the receptor protein which stimulates the binding of a heterotrimeric G-protein to the GPCR. Heterotrimeric G-proteins are composed of alpha, beta and gamma subunits. These subunits are triggered to interact with the receptor [2]. Once a receptor is activated (Figure 1) the GDP which is bound to the Gα-subunit is exchanged to GTP. The Gα-subunit dissociates from the Gβγ dimer resulting in two functional subunits (Gα and Gβγ dimer) signaling to downstream effectors like adenylyl cyclases or guanylcyclases which are responsible for cyclization of ATP/GTP to cAMP/cGMP. Phosphodiesterases then hydrolize cAMP once a threshold has been reached. Finally, cAMP-dependent protein kinase A (PKA) or cGMP-dependent protein kinase G (PKG) is activated.

Depending on the sequence and the functional similarity, Gα-subunits consist of four different subfamilies [3], i.e., (Gα_s_, Gα_i/o_, Gα_q11_ and Gα _12/13_). Within the Gα_s_ subfamily there are two groups i.e., the Gα_s_ (stimulatory) and the Gα_ol_ (olfactory). Gα_s_ is expressed in all types of cells while Gα_ol_ is expressed in olfactory sensory neurons [4]. Gα_i_ is the largest and most diverse family. Gα_t1_ (t means transducin) is expressed in the rod cells in the eye, while Gα_t2_ is expressed in the cone cells of the eye [5]. In humans, Gα_q_ is ubiquitously expressed and Gα_12/13_ occurs in all types of cells.

In contrast to the impressive progress in understanding cyclic nucleotide signaling in the mammalian host, research on this topic has been delayed in apicomplexan parasites and kinetoplastids. One reason might be the recently completed genomes of these parasites which revealed very different components of the pathways. In particular, subunits of canonical G-proteins are absent in their genomes. Moreover, it has to be clarified whether the few database entries encoding proteins with serpentine motifs might have GPCR-like functions. Instead, secondary effector proteins are present which differ considerably compared to their human counterpart. Apicomplexan adenylyl cyclase (AC) and guanylyl cyclase (GC) are bifunctional enzymes, harbouring either a putative ion channel (AC) or a P-type ATPase-like domain (GC) alongside the catalytic region [6]. Kinetoplastids contain only a single highly variable transmembrane domain and a catalytic domain, consistent with multiple receptor-activated cyclases [6]. Phosphodiesterases (PDEs) are highly conserved in the Apicomplexa and kinetoplastids but are almost insensitive to the mammalian inhibitors. PKA is a well characterized serine threonine ACG kinase in *Plasmodium* and gains a role as a druggable target since it is essential in almost every stage of parasite development [7]. The apicomplexan PKG has structural elements and biochemical properties that distinguishes it from the human orthologues. There are four cGMP binding domains of which only three are functional [8]. Plasmodial PKG has been successfully validated by a pyrrole, the 4-[2-(4-fluorophenyl)-5-(1-methylpiperidine-4-yl)-1*H*-pyrrol-3-yl]pyridine [9] and even the more effective imidazopyridines [10].

Moreover, apicomplexan parasites and kinetoplastids possess a set of small GTPases to infect the host cell in the absence of canonical G-proteins and putative GPCRs. Most of these small, identified GTPases belong to the Ras superfamily [11] This superfamily consists of five different classes i.e., Ras (rat sarcoma oncoproteins), Arf (ADP-ribosylation factors), Ran (Ras related nuclear GTPases), Rab (ras related proteins in brain) and Rho (Ras homologous GTPases) acting as molecular switches. Mostly represented in apicomplexan parasites and kinetoplastids are the Rab proteins controlling intracellular, vesicular transport. A variety of functions can be attributed to the Ras superfamily like the control of gene expression and cell proliferation, vesicle coating, nucleo-cytoplasmatic transport and regulation of cell cycle progression. Cycling of these GTPases occurs between an inactive GDP-bound form and an active GTP-bound form which is controlled by regulators i.e., activators like guanine nucleotide exchange factors (GEFs) (more than 60 are known in the human counterpart) and inhibitors like GTPase-activating proteins (GAPs) (more than 70 are known in humans). More than 100 target/effector proteins have been described [11]. Mostly Rho GTPases are involved in host pathogen interaction by controlling innate and adaptive immune responses [12]. Alternatively, parasites have also developed strategies to modify host GTPases to ensure their own proliferation by killing the eukaryotic host cell. In this context, it is an interesting observation that interferone-regulated GTPases in the host cell can be directly inactivated by the parasite. However, in case of some highly variable surface glycoproteins (VSG), T-cell receptor mediated and independent responses in the host are involved.

Based on these scientific findings it is difficult to understand why no antiparasitic drug targeting small GTPases, G-proteins and putative GPCRs has yet reached the clinical phase although apicomplexan parasites and kinetoplastids represent the most important human infectious diseases. One important reason is the fact that canonical cyclic nucleotide pathways which are used by the parasite exist in the host cell and thus contribute to pathogenicity. In conclusion, a fixed drug combination with highly selective compounds may be necessary, one to specifically eradicate the parasite and the other to suppress the canonical pathway in the human host which is utilized by the parasite. This review focuses on small G-proteins, in particular small GTPases and individual targets present in cAMP-signaling pathways in the absence of canonical G-proteins in the parasite and the mammalian host. Current knowledge about proteins that might have GPCR-like functions is presented. Secondly, the current data for target evaluation and identification of small molecules are critically discussed.

## 2. cAMP-Signaling in Apicomplexan Parasites without Canonical G-Proteins and G-Protein-Coupled Receptors (GPCRs): A Comparison to the Human Counterpart

### 2.1. Non Canonical G-Proteins and Putative GPCRs in Plasmodium

Research on GPCR-mediated signaling pathways in *Plasmodium* has just started over the last decade. During infection the malaria parasite has to adapt to different environmental changes in the human host i.e., the pre-erythrocytic stage in the human liver and the erythrocytic blood stages. Within the sexual stage in the mosquito, ookinetes develop in the mosquito midgut to form an oocyst which bursts to release sporozoites into the salivary glands [13].

In 2009 the newly founded malaria signaling consortium [14] began to study the molecular mechanisms which enable the parasite to sense and adapt to the intra- and extra-cellular requirements, i.e., invasion of the hepatocytes in the human liver, the erythrocytic stages in the human host and the sexual development in the *Anopheles* mosquito. In sum, the current results provide evidence that the cyclic nucleotides cAMP or cGMP are essential during stage conversion of the asexual, intraerythrocytic stages to the presexual stages [15]. In particular these cyclic nucleotides are required in exflagellation during male gametogenesis where a gametocyte is converted to a male gamete in the mosquito.

However, knowledge about the first part of the pathway, in which a GPCR-mediated signal might be transferred via heterotrimeric G-proteins to adenylyl cyclase is scarce in *Plasmodium*. To understand the pathogenesis of a malaria infection one has to consider both molecular mechanisms of signaling in the human host and the parasite. Signal transduction occurs between the human erythrocyte and the parasitophorous membrane. Studies began in the enucleated human erythrocyte where a set of proteins was identified which were involved in signaling [16]. For an outside-in, signaling in infected erythrocytes membrane receptors such as purinergic receptors were demonstrated to be responsible [17]. Secondly, extracellular ATP increased cation fluxes in human erythrocytes [18] and ion channels were activated during infection with malaria parasites [19]. Thirdly, it was shown [20] that signaling via the erythrocyte-beta-2-adrenergic receptor and heterotrimeric guanine nucleotide-binding protein (Gα) regulated the entry of the human malaria parasite *Plasmodium falciparum*. Meanwhile there is evidence that cAMP which is not localized to a signal transduction pathway can activate exchange proteins (EPAC) that inhibit ATP release [21].

Collectively, erythrocytic G-proteins reside at the cytoplasmic face of the cellular plasma membrane, where they can couple with a variety of transmembrane receptors to transduce extracellular signals to proteins (Gα_s_) and thus facilitate invasion of the parasite [22].

Further results revealed that the erythrocyte host G-alpha-s subunit (Gαs) which is located at the cytoplasmatic face of the plasma membrane is functional and promotes invasion of the parasite into the erythrocyte [23]. Supplementation of in vitro *P. falciparum* cultures with propranolol, an antagonist of the G-protein-coupled β-adrenergic receptor inhibited intracellular parasite growth. Thus inhibition of signal transduction via the Gαs in the erythrocyte membrane reduced invasion of the parasite [23]. These results led to the conclusion that signaling in *Plasmodium* is unlikely to be via heterotrimeric G-proteins from the parasite and depends exclusively on GPCR-mediated signaling in the erythrocyte of the human host. Hitherto, there is no genomic evidence that canonical heterotrimeric G-proteins exist in *Plasmodium*. Apart from the genomic data, biochemical experiments with mastoparan 7 and cholera-toxin (CTX) [24] were performed. CTX stimulates ADP-ribosylation of the Gs alpha-subunit [25] while mastoparan 7 increases GTPase activity. Taken together, from these experiments the authors concluded that only mastoparan 7 significantly increased cAMP levels while cholera toxin induced commitment to gametogenesis without heterotrimeric G-proteins.

However, four putative entries of GPCR-like receptors currently exist in the *Plasmodium* database (PlasmoDB), i.e., two encoding for serpentine receptors and one encoding a GPCR-like receptor occurring in *P. falciparum*, *P. reichenowi* and *P. vivax Salvador* strain-1. One of the putitative GPCR encoding nucleic acid sequences has been recently characterized [26]. This serpentine receptor protein SR25 functions as a monovalent cation sensor capable of modulating Ca^2+^ signaling in the parasite. A shift of high to low K^+^ concentration triggers an increase in Ca^2+^ concentration which can either be blocked by phospholipase C inhibition or by depletion of the parasite’s internal calcium pool. Deletion of the SR25 receptor protein leads to insensitivity to hyperosmotic stress, decreased parasitemia, and metacaspase gene expression [26] It remains an open question whether this putitative GPCR might be a druggable target in *Plasmodium* since deletion of the SR25 receptor does not eradicate the parasite. However, it would be of considerable interest to delineate in which developmental stage this receptor is functionally active. Three other database entries for putitative GPCRs still lack a proof of functionality and their ligands are yet unknown.

### 2.2. Small GTPases in Plasmodium May Substitute Canonical G-Proteins

Instead of heterotrimeric G-proteins, a small set of Rab GTPases and a Ras GTPase was identified in *Plasmodium.* Rab GTPases are involved in the regulation of targeting and fusion of transport vesicles within the secretory and endocytic pathways [27] (Langsley et al., 2008). Most of the apicomplexan proteins have predicted signal peptides which direct them to the cytosol of the red blood cell (RBC), to the apicoplast and Maurer’s Clefs. In contrast, signal peptides of proteins that are directed to secretory organelles like rhoptries, micronemes and dense granules during erythrocyte invasion remain to be identified.

Rabs are associated with the cytoplasmic surface of a particular membrane compartment in the cell. However, they can also exist as a soluble protein in the cytoplasm like the GDP-bound form of Rab which is soluble in the cytoplasm as a complex with guanine nucleotide dissociation inhibitors (GDI). Rabs become associated with membranes when a GDI displacement factor exposes a prenyl group covalently linked to the C-terminal end of the Rab protein which then inserts into the membrane. Once the GDI is released, a guanine nucleotide exchange factor (GEF) on the membrane activates the Rab by exchanging GDP to GTP. Activated Rabs interact with a range of different partners. In addition to vesicle docking, Rabs are also involved in vesicle formation and movement.

There are 11 different Rab isoforms in *Plasmodium* [28]. Two small, monomeric GTPases Rab1A proteins i.e., PfRab1A and PfRab1B were further characterized in *Plasmodium* which regulate vesicular traffic from the endoplasmatic reticulum (ER) to the rhoptries in late-phase schizonts [29]. While PfRab1A is a paralogue of Rab1 found in the chromalveloates, a phylogenetic group within the Apicomplexa, the PfRab1B is more closely related to other organisms.

A unique myristoylated Rab5B GTPase from three occurring in *Plasmodium* was identified that was targeted to the food vacuole of the parasite to import nutrients from the RBC for an abundant food supply from the hemoglobin of the host cell [30]. In conclusion, mostly Rab proteins which belong to the Ras superfamily were identified in *Plasmodium* that regulate the budding and fusion of intracellular vesicles from donor to acceptor membranes.

A recent screening for G-protein orthologues in *Plasmodium* resulted in the identification of a non-canonical Ras-like GTPase (acronym PfG) [31]. PfG might represent a multi-enzyme complex (molecular weight 109 kDa) which is localized to the cytosol and has an EngA domain present in bacterial GTPases. PfG is highly divergent from any of the known Gα_s_ human subunits and still has an unknown function.

### 2.3. A Step towards Selective Inhibitors of Heterotrimeric G Proteins in Mammalian Cells

Thirty to forty percent medical drugs are commercialized that target mammalian GPCRs. In contrast, only a few pharmacological inhibitors had been characterized that target mammalian, heterotrimeric G-proteins. However, recent studies are encouraging with respect to fundamental research on GPCR pharmacology and the potential application in GPCR-related disorders [32]. Nevertheless, only one of them has ever reached clinical trials. The first small molecules identified were bacterial toxins like CTX, *Bordella* pertussis toxin (PTX), heat-labile enterotoxin of *Escherichia coli*, and *Pasteurella multocida* toxin (PMT). Their mode of action is based on the chemical modification of critical residues of the Gα- subunit [33] with effects on GTP hydrolysis and the interacting GPCR. One of the oldest molecules that targets the Gα-subunit is suramin which had been originally designed for the treatment of African trypanosomiasis. The large structure of suramin prevents the association with the β- and Χ-subunits and in consequence the coupling of the GPCR [34]. In the 2000s two imidazopyrazines BIM-46174 (7-[2-amino-1-oxo-3-thio-propyl]-8-cyclohexylmethyl-2-phenyl-5,6,7,8-tetrahydro-imidazo [1,2a]-pyrazine) and BIM-46187 (8S,8′S)-7,7′-[Dithiobis[(2R)-2-amino-1-oxo-3,1-propanediyl]]bis[8-(cyclohexylmethyl)-5,6,7,8-tetrahydro-2-phenyl-imidazo[1,2-a]pyrazine were shown to inhibit the Gαs. BIM-46174 was found selectively to inhibit the cAMP increase induced by CTX via the activation of Gαs [35]. In addition it had anticancer activity. Interestingly, the compound had further downstream effects when it was applied in a GPCR/G protein transient expression system in cos cells [36].

Two cyclic depsipeptides YM254890 and FR900359 from *Chromobacterium* and the tropical plant *Ardisia crenata,* respectively, were selective inhibitors of the Gαq subunit by preventing GDP release [37]. In vivo and ex vivo studies have used FR900359 to reveal the involvement of the Gαq- mediated signaling pathway in various pathophysiologies including obesity and asthma [38,39]. While PTX was widely used to block Gαi, quinazoline derivatives have been recently demonstrated to inhibit Gαi selectively [40].

The GβΧ subunits are also attractive targets since they interact with multiple effectors i.e., adenylyl cyclase, phospho-inositide-3-kinase Χ, phospholipase C. These signaling effectors mediate key responses such as neutrophil chemotaxis and vascular cell proliferation [41,42]. The most important pharmacological tool acting on the GβΧ subunit is the GRK2ct. The GRK2 stands for G-protein-coupled receptor kinase 2 which has the GβΧ-subunit binding domain. The GRK2ct consists of a C-terminal 194 amino acid peptide of GRK2 responsible for the binding of the GβΧ-subunit and interfering with GRK2 activity by disrupting the membrane translocation [43] of the βΧ subunits. These findings led to the development of small molecules interfering with GβΧ association. A critical peptide SIGK was identified [44] and a potent compound M119 discovered with high apparent affinity for Gβ1γ2 (IC_50_ = 200 nM). In conclusion, pharmacological inhibition of the different mammalian Gα or the GβΧ subunits might be a novel strategy in drug discovery since it would cause an interruption of host specific G-protein signaling which is responsible for invasion and proliferation of the parasite.

## 3. Toxoplasma Gondii Uses the GPCR-Signaling Network and GTPases from the Infected Host Cell Besides Its Own Small GTPases

*Toxoplasma gondii* is a ubiquitous protozoan parasite found in a wide variety of hosts, including humans. It infects 30% of the human population [45]. It appears in three different forms i.e., oocysts, tachyzoites and bradyzoites. Transmission occurs through the feces where oocysts reside. The tachyzoite form is crescent-shaped and is usually found in intracellular clusters of 8 to 32 parasites and is the rapidly replicating form. The tachyzoites are responsible for tissue damage and the infection of the fetus in pregnant women. Tachyzoites develop into bradyzoites in particular in tissues of the muscle or the central nervous system (CNS) due to the host response immune reaction. Tachyzoites activate a potent host immune response that eliminates most of the parasites. Some tachyzoites, however, escape destruction and convert back into bradyzoites.

Bradyzoites can be ingested in meat and when taken up by the human host they convert to tachyzoites. In *Toxoplasma gondii* knowledge about signaling via G-protein mediated GPCRs is still rudimentary. Hitherto, there are no reports about canonical G-proteins in the *Toxoplasma* database (http:/toxodb.org/toxo/) except for proteins containing repeats of motifs from the β subunits of canonical G-proteins. Hence, there is only one database entry from *Toxoplasma* encoding a rhodopsin-like GPCR transmembrane domain protein. However, functional data for this receptor protein are still missing. Therefore, this database entry has to be considered critically. Moreover, there is a lack of knowledge about GTPases in *Toxoplasma gondii*.

Apicomplexan parasites like *Toxoplasma gondii* are dependent on the GPCR signaling network from the human host [45]. Secondly, parasite-induced host cell cytolysis has been suggested to be a two-step, Ca^2+^-dependent process [46]. This two-step process comprises ion loss of the host cell [47] and membrane poration. Previous results have implicated that three different parasitic proteins are involved in this process, i.e., a perforin-like protein [48], a kinase [49] and proteases that have not been further characterized [50]. However, recent data pinpoint [45] that host cell cytolysis is controlled by Gα_q_, phospholipase C (PLC), and protein kinase C (PKC). Gα_q_-coupled signaling results in protein kinase C (PKC)-mediated loss of the host cytoskeletal protein adducin and catastrophic influx of Ca^2+^ through the cation channel TRPC6 which activates calpain to proteolyze the host cytoskeleton. Thus targeting proteins involved in host cell cytolysis might be an alternative, antiparasitic strategy that should be explored. Indeed, it was shown that a mammalian PKC-inhibitor Gö6976 (5,6,7,13-Tetrahydro-13-methyl-5-oxo-12*H*-indolo[2,3-*a*]pyrrolo[3,4-*c*]carbazole-12-propane-nitrile) limited *T. gondii* parasitic burden in vivo in spleen [51] of the infected mice.

In addition, a few host GTPases have been identified that play an important role in *Toxoplasma gondii* infection. Interestingly, as an intracellular parasite *Toxoplasma* replicates in a parasitophorous vacuole (PV) that interacts with mammalian host organelles and intercepts Golgi Rab vesicles to acquire sphingolipids [52]. Thus, interrupting this vesicular pathway might be a successful strategy to prevent *Toxoplasma gondii* infection. In *Toxoplasma gondii* immunity related host GTPases (IRGs) are also recruited to pathogen-containing vacuoles, where they are important for their disruption resulting in cell-autonomous clearance. However, the suppressive mechanism was unclear. Meanwhile, it could be shown that Rab GDP dissociation inhibitor α (RabGDIα) acts as a suppressor of IFN-γ–inducible host GTPases, such as Gbp2 and Irga6. RabGDIα deficiency resulted in enhanced IFN-γ–mediated *T. gondii* clearance in vitro and in vivo [53]. These observations can be attributed to a strong interaction between immunity-related host GTPases (IRG) and rhoptry (Rob5) pseudokinases expressed by the parasite [54] having two effects: (i) prevention of oligomerization of the IRG, and (ii) change in the orientation of two threonine residues that are targeted by the *Toxoplasma* Ser/Thr kinases, ROP17 and ROP18.

## 4. The Non-Intracellular Parasite *Cryptosporidium* Can Manage Its Proliferation without G-Proteins and GPCRs

Human cryptospoiridiosis is caused by *Cryptosporidium hominis* and *Cryptospridium parvum* and causes a self-limiting diarrhea in healthy people [55]. While transmission of *Cryptosporidium hominis* exclusively occurs in humans, *Cryptosporidium parvum* infects both animals and humans. However, in immunodeficient people i.e., HIV-1 patients and small children, it leads to prolonged and persistant life-threatening diarrhea with an impact on the biliary tree and the respiratory tract [55]. *Cryptosporidium* infections start with a sporulated oocyst which is transferred by contaminated food and water. However, it is also possible that *Cryptosporidium* can be transmitted by inhalation. While sporozoites are released in the intestine, they are able to infect and reproduce in the epithelial cell of the gastrointestinal tract or respiratory organs. During the asexual cyle they undergo schizogony or merogony before sexual multiplication forming microgamonts and macrogamonts starts. Finally, fertilization of the macrogamonts leads to the formation of a sporulating oocyste which can be transmitted faecally or orally.

While there are no reports about canonical G-proteins in *Cryptosporidium*, there are several database entries for small GTPases in different *Cryptosporidium* species. The most promising protein sequence encodes a putative, developmentally regulated protein, protein 2 from *Cryptosporidium muris*. This protein sequence shows 54% amino acid identity to a developmentally regulated GTP-binding (DRG) protein from yeast and 61% amino acid identity to the human paralogue. DRGs are highly conserved GTPases that associate with DRG family regulatory proteins (DFRP). The resulting complexes have been shown to participate in eukaryotic translation [56]. Thus, it may be tempting to speculate that differentially expressed GTPases might function as a substitute for canonical G-proteins. Notably, cholangiocytes, a line of epithelial cells in in bile ducts, express a small Ras GTPase, that mediates cholangiocyte proinflammatory cytokine production and induction of cholangiocyte proliferation after infection with *Cryptosporidium parvum* [57].

To date, there has been only one report about a putative GPCR coding sequence in the *Cryptosporidium* database (CrypDB). However, this report is considered to be mysterious although the 486 amino acids encoded protein has a high score to a Rhodopsin-like GPCR transmembrane protein. The seven transmembrane alpha-helical domain is present in GPCRs and highly conserved from nematodes to humans [58]. Collectively, these data may imply that *Cryptosporidium* is not strictly dependent on the host GPCR signaling network. Almost 10 years ago, a key protein Ran, driving nuclear transport, was identified in the *Cryptosporidium* genome [59]. However, a characterization of this Ras GTPase is still missing.

## 5. Non-Canonical Camp-Signaling Pathways in the Kinetoplastids: Trypanosomes and Leishmania Have a Variety of GTPases with Specialized Functions

Trypanosomiasis comprises the name of distinct disease patterns in vertebrates. In human it appears as African trypanosomiasis [60] and as Chagas disease. African trypanosomiasis has two different stages. In the first stage the parasite enters the peripheral circulation before it crosses the blood–brain barrier in the second stage [61] and spreads into the central nervous system. If the patients are not treated, death will occur within months. The life cycle of the transmitted trypomastigotes in the human blood stages starts after the bite of the tsetse fly. In turn, trypomastigotes develop in the midgut of the fly to the procyclic forms and subsequently to epimastigotes. Finally, epimastigotes are transformed to the metacyclic forms in the salivary glands.

In contrast, *Trypanosoma cruzi* is transmitted to humans by blood-sucking triatomine bugs causing Chagas disease in Central and South America [62]. The disease appears in an acute and chronic stage. The acute phase most often appears mild and parasites may occur in the blood circulation. After several weeks or months of infection, most patients enter the chronical phase showing life-threatening medical symptoms including heart rhythm abnormalties or heart dilations with sudden death. Chemotherapy for human African trypanosomiasis (HAT) is still limited (see chapter 8).

Instead of canonical G-proteins small GTPases were identified in trypanosomes. Ras-like GTPases are particularly prevalent in vesicular transport. A Ras-like GTPase was identified in *T. brucei* with an unsual mechanism of action that involves proteolysis of the nascent protein and membrane targeting via phosphoinositol-3-phosphate (PI3P) [63]. A Rab21 paralogue from *T. brucei* functions in intermediate steps of the endocytic pathway [64]. Endocytosis is one of the mechanisms that the parasite uses to evade the host innate immune response. Trypanosomes in the bloodstream of infected animals take up various particulate and soluble substances in vesicles morphologically similar to the clathrin-coated vesicles found in other eukaryotic cells for their growth. These vesicles bud only from the membrane of the flagellar pocket, a deep invagination of the plasma membrane where the flagellum leaves the cell, and discharge their contents into an intracellular tubular system.

More recently, it has emerged that endocytosis is the major mechanism for the uptake of first-line trypanocides which offers a potential way to deliver therapeutics to the parasite in a highly efficient manner [65].

Another set of small GTPases is involved in the motility of trypanosomes, i.e., the interflagellar transport. The GTPase IFT27 [66] is required for the association to the IFT (interflagellar complex). The small GTPase Arl6 from *T. brucei* which is responsible for the length of the flagellum in the human bloodstream stages was recently crystallized with the bound non-hydrolysable GTP analog GppNp [67]. These results revealed significant differences to the human paralogue i.e., a lack of a key glutamine in the active site that activates the nucleophile during GTP hydrolysis in other small GTPases. Furthermore, the trypanosomal protein is shorter at the N-terminus. These structural differences might support the discovery of pharmacological, selective inhibitors. Another Ras-related GTPase belonging to the RJL family has been described in *T. cruzi* [68]. This Ras GTPase is mainly found in the GTP-bound stage and is localized closely to the flagellar apparatus. TcRjl function is required for cell growth and its overexpression blocks *T. cruzi* metacyclogenesis.

One important protein for the actin-dependent invasion of external amastigotes of *Trypanosoma cruzi* into a host cell is Rac1, a member of Rho GTPases [69]. Two small GTPases Sar1 and ARF1 were identified during Golgi biogenesis in *T. brucei* as a model organism [70]. Collectively, these data suggest that small GTPases fulfill plenty specialized functions in trypanosomes.

Although there is no genomic evidence for GPCRs in the genome of trypanosomes, a prostanoid-like receptor has been identified in *T. cruzi* [71]. This thromboxane A_2_ derived receptor [72] responds to thromboxane A_2_ mimetics and modulates vasculopathy in Chagasic cardiomyopathy.

The receptor activates MAP kinase activated pathways and localizes to different parts of the flagellum. Although there are similar glycosylation patterns to that of its human paralogue this receptor is unique for *Trypanosoma cruzi* [73] and might be an interesting candidate for target evaluation.

Leishmaniasis is a tropical neglected diesase that is caused by different *Leishmania* species which are transmitted by sandflies (phlebotomes). The disease appears mainly in two distinct forms, i.e., cutaneous leishmaniasis [74], which causes skin sores, and visceral leishmaniasis [75], which affects several internal organs (usually spleen, liver, and bone marrow). Some people have a silent infection but most of them develop clinical evidence. For cutaneous leishmaniasis the sores may start out as papules (bumps) or nodules (lumps) and may end up as ulcers. In the case of visceral leishmaniasis, patients usually have fever, weight loss, enlargement (swelling) of the spleen and liver. According to World Health Organization (WHO) reports for 2018, estimated cases of visceral leishmaniasis are 0.4 million to 1.2 million per year while 1.2 million people or even more are infected by cutaneous leishmaniasis. Human infection is caused by about 21 species. These include the *L. donovani* complex, the *L. mexicana* complex, *L. tropica* and *L. major*. Transmission of leishmaniasis is performed by a bite of female sandflies. Promastigotes are phagocytized by macrophages and transformed into amastigotes in the tissue. When taken up by a sandfly, they are retransformed into promastigotes in the gut before they migrate into the proboscis.

Hitherto, there have been no reports about heterotrimeric G-proteins binding to GPCRs in *Leishmania* species.

In parallel to the situation in trypanosomes, a variety of GTPases exists in *Leishmania* with functions in intracellular trafficking, secretory pathways, endocytosis and pathogenesis. Some of the most important data are reviewed herein.

A monomeric GTPase Rab1 homologue has been recently characterized in the secretory pathway of *Leishmania* [76]. Unexpectedly, transgenic parasites expressing a GTPase-deficient dominant positive mutant of Rab1 or a GDP-locked dominant negative mutant of Rab1 do not disrupt trafficking or localization of the hemoglobin receptor in *Leishmania*. However, secretion is impaired in both mutant lines suggesting that Rab1 does not control trafficking. In contrast, the GTPase Sar1 from *Leishmania* regulates secretion and trafficking of the virulence factor gp63, a metalloprotease [77] and is essential for parasite survival. Interestingly, Rab5 isoforms from *Leishmania* were demonstrated to regulate fluid-phase or receptor-mediated endocytosis [78]. A diagnostic role and possible clinical use was attributed to Rab6 as a key regulator of intracellular vesicular transport and trafficking [79]. An antibody raised against the Rab6 protein detected the protein in sera from patients with Indian visceral leishmaniasis.

CD40, a transmembrane costimulatory receptor that is expressed in macrophages induces activation of different Ras-isoforms. The receptor has dual function in signaling. It can induce either Raf-MEK-ERK-1/2-mediated anti-inflammatory IL-10 production or P13K-MKK-p38MAPK-mediated proinflammatory IL-12 production. Depending on the Ras isoform in the domain that is induced, the outcome of an infection by *Leishmania major* causing cutaneous leishmaniosis can be different since the parasite is able to switch the signal. CD40-induced IL-10 promotes *Leishmania* infection whereas CD40-induced IL-12 protects hosts from the infection. It was demonstrated that CD40 induced N-Ras activation resulted in a reduced *Leishmania* infection [80].

## 6. Metabolism of cAMP in the Apicomplexa

Hitherto, four cyclic nucleotide phosphodiesterases (PDE_α-δ_) were identified in *Plasmodium*. PDE_α_ and PDE_β_ are expressed in the late erythrocytic stages while expression of PDEγ and PDE_δ_ appears in sporozoite and gametocyte stages, respectively. PDE_α_ is not essential in in vitro blood stages and sensitive to zaprinast, (1,4-Dihydro-5-(2-propoxyphenyl)-7*H*-1,2,3-triazolo(4,5-d)pyrimidin-7-one) a mamma-lian PDE inhibitor [81]. Zaprinast turned out to be a low micromolar inhibitor of recombinant and native PDE activity and blood stage growth [82]. Analogues of taldalafil, a human PDE5 inhibitor showed even better results for the EC_50_ which were in the submicromolar level [83]. In a second drug to genome to drug approach, the authors substituted the piperonyl group in taldalafil and introduced a dimethoxyphenyl group to achieve better selectivity [84] for the parasitic enzyme. Deletion mutants were obtained for PDE_δ_ which was dispensable in the blood stages but essential in the exflagellation of male gametocytes [85]. Interestingly, PDE_δ_ mutants showed a reduced transmission rate after infecting mice to establish a blood-stage infection but a strong expression in sporozoites [86]. Since there is a lack of sporontocides, PDEΧ might be a drug target candidate that needs further characterization. Curiously, *T. gondii* has 18 potential cyclic-nucleotide phospho-diesterases (http://toxodb./org/toxo/). It is tempting to speculate that they might be expressed in different developmental stages of the parasite. There are no experimental data about the application of mammalian inhibitors like zaprinast or its derivatives on these 18 potential cyclic-nucleotide phosphodiesterases.

## 7. cAMP in Apicomplexan Parasites Activates the Guanine Exchange Factor (GEF) EPAC and Rap1

Adenylylcyclases [87] and guanylylcyclases [88] have been identified in *Plasmodium* together with phosphodiesterases [89] and their cAMP/cGMP dependent-kinases [90]. Since their structural peculiarities and their biochemistry have been extensively reviewed [90], the focus here will be on the small GTPase Rap1 (rhoptry associated-protein-1) which is activated by cAMP as the second messenger. This effect is mediated by the guanine nucleotide exchange factor (GEF) EPAC, which is directly activated by cAMP and independent from protein kinase A. Thus Rap-1 has an important function in invasion of the RBC.

Two adenylyl cyclases and two guanyl cyclases occur in *Plasmodium*. Adenylyl cyclase alpha (*Pf*AC_α_) seems to be involved in sporozoite exocytosis while PfAC_β_ is essential for the blood stages as shown in a rodent model by deletion experiments [90]. Moreover, the authors hypothesized that *Pf*AC_β_ acts as a pH sensor. Both proteins differ in their structural architecture. *Pf*AC_α_ contains six predicted transmembrane domains with significant homology to voltage-gated K^+^ channels and a single carboxy-terminal catalytic domain homologous to soluble adenylylcyclases (sAC). In contrast, *Pf*AC_β_ has no predicted transmembrane regions and possesses two sAC-like AC catalytic domains. *Pf*AC_β_ is an orthologue of the bicarbonate-sensitive adenylyl cyclases. To date, two pharmacological inhibitors i.e., KH7 (*E*)-2-(1*H*-Benzo[d]imidazol-2-ylthio)-*N*′-(5-bromo-2-hydroxybenzylidene) propanehydrazide) and 2-catechol estrogens (CE) [15] showed inhibition of PfAC_β_ in the micromolar range. The inhibitory effect might be explained by the fact that either the imidazole- or the catechol- structure can complex the metal ion (magnesium) in the catalytic site.

During egress and invasion of merozoites PfACβ triggers cAMP levels fostering the EPAC pathway which in turn leads to phosphorylation of Rap-GDP to Rap-GTP [91] (Figure 1). Rap-GTP activates phospholipase C (PLC) to produce inositol triphosphte (IP_3_) that binds to the IP_3_ receptor (IP_3_R) on the endoplasmatic reticulum (Figure 1). This leads to a release of calcium ions. Calcium ions bind to calcineurin and activate calcium-dependent protein kinase 1 (CDPK1) (Figure 1) required for the discharge of microneme and rhoptry to facilitate invasion of the parasite. In addition to Plasmodium, a Rap-1 protein has been characterized in the Apicomplexan Babesia motasi which infects sheep [92] and horses [93]. To date, no chemical inhibitor was identified that inhibits Rap-1 from the parasites directly.

Initially, Rap-1 was identified as a ras suppressor protein able to revert the morphological phenotype of Ras-transformed fibroblasts. Meanwhile, it was demonstrated that Rap-1 is activated by a variety of different receptors. Several messengers like calcium, diacylglycerol and cAMP mediate this effect. Inactivation is performed by specific GTPase-activating proteins (GAPs), one of which is regulated through an interaction with Galpha_i_.

Currently, there have been no reports about Rap-1 orthologues in the database of *Toxoplasma gondii* and *Cryptosporidium* yet, although a putative adenylyl cyclase β from *T. gondii* encodes a unique protein of 2400 amino acids with a conserved, catalytic adenylyl cyclase class III domain. Separate loci, encoding guanylyl cyclases have also been identified in the genome of *T. gondii* with a typical, conserved domain for adenylyl- and guanyl cyclases. The amino acid identity corresponding to the *Cryptosporidium* paralogues is approximately 48% and to the rodent *Plasmodium* species 36%.

cAMP is necessary for the differentiation of the rapidly replicating tachyzoites (infective form) to the slowly proliferating bradyzoites that persist as a latent infection [94]. In parallel to *Plasmodium*, a protein (ROP4) was identified in *T. gondii* which is located on the outer face of the rhoptries that are responsible for parasitic invasion [95] (Mueller et al., 2016). The association with the rhoptries is dependent on the presence of an interacting partner, the armadillo repeat-only protein (ARO) from the parasite. The ROP4 protein is phosphorylated in the infected cell. This step is performed by parasite or host cell kinases which are induced by host cell factors [96].

## 8. cAMP Signaling in the Kinetoplasids: Small GTPases Control Host Cell Invasion

Despite recent advances in drug research, finding a safe, effective, and easy to use chemotherapy for HAT caused by *Trypanosoma* ssp. still remains a challenging task [97]. Pentamidine and suramine can be administered only in the first stage of infection of HAT. The second stage drugs melarsoprolol and eflornithine have disadvantages of their own. Melarsoprolol is toxic causing encephalopathy, and eflornithine is laborious to administer and lacks efficacy against *T. brucei rhodesiense* (East African Sleeping sickness). There might be an an opportunity in targeting downstream effectors of a cAMP-signaling pathway with remarkable features in trypanosomes. Although there is inadequacy of current treatments which should rather speed up drug development, new drugs have been slow to emerge.

The importance of cAMP as a second messenger molecule in trypanosomes is reflected by the high number of genome-encoded adenylate cyclases (ACs) with more than 80 genes and pseudogenes. The first adenylate cyclase X-ray structure was obtained from *Trypanosoma brucei* causing African trypanosomiasis [98]. This AC belongs to the type II class of ACs which exclusively occur in protozoa. They contain an extracellular domain, for putative receptor binding, a single transmembrane domain span, and a cytosolic, catalytic region. ACs are structurally different in comparison to their mammalian counterparts. While the mammalian protein has 12 transmembrane domains, *Trypanosoma brucei* has only one. However, the basic catalytic mechanism seems to be conserved between them. Trypanosomes possess ACs with a variable large extracellular N-terminal domain and an insertion in the G-protein-binding domain although G-proteins are absent. However, the ligands binding to the N-terminal domain remain to be identified. To date, no experimental report for an AC from *Trypanosoma cruzi* has been published. 

In their genome, trypanosomes contain multiple expression sites (ESs) for a highly variable surface glycoprotein (VSG) to evade the host immune response. Downstream of the ESs, a set of genes called expression site-associated genes (ESAGs) are cotranscribed. These genes modulate the host innate immune response [99]. It was shown by RNAi that the small GTPase Rap-1 controls VSG expression in blood stream forms (BF) and at the insect stage (procyclic form) [100]. Targeting these genes which are highly expressed in the blood stream form might be a powerful tool for drug discovery.

Collectively, it is now evident that instead of cAMP the products of cAMP hydrolysis are responsible for differentiation in trypanosomes [101]. This does not rule out the involvement of canonical cAMP signaling components like ACs since they may be necessary for initial cAMP production.

There are four groups of cAMP-dependent phosphodiesterases in trypanosomes. Each group has one or two representatives. Most of them are structurally similar to human PDEs. The first crystal structure of phosphodiesterase B1 (PDEB1) was obtained from *Trypanosoma brucei* [102]. The structure confirmed the presence of a parasite-specific pocket between amino acid residues Gln874 and Tyr845 and is absent in the human orthologue. This property has been shown to be useful for the design of specific lead structures and inhibitors. In human PDEs the pocket is gated by two large residues while smaller residues are found in the parasite orthologue. These findings resulted in an approach of targeting the P-pocket with hPDE inhibitors developing more specificity for the parasitic enzyme on the basis of fragment growing [103]. The resulting catechol pyrazolines act as potent TbrPDEB1 inhibitors with IC_50_ values down to 49 nM. The compounds also block parasite proliferation. Knockdown experiments of PDEB1 and PDEB2 in mouse models provided clues that PDEs are necessary for motility and virulence [104].

During infection, *Leishmania* parasites arrest in phagolysosomes in the host cell to avoid transport to the lysosomes. *Leishmania donovani* specifically upregulates the expression of Rab5a by degrading c-Jun via their metalloprotease gp63 to downregulate the expression of miR-494 in THP-1 differentiated human macrophages [105].

In *Leishmania* species, downstream effector proteins of a cAMP-regulated pathway are present. However, it is not clear whether cAMP activates the EPAC pathway to phosphorylate Rap-1. More than 10 stage-regulated adenylate cyclases were identified in *Leishmania* species. Previously, two receptor adenylate cyclases from *L. donovani* (LdracA and LdracB) were analyzed which form a part of a cluster of five similar genes [106]. They were observed to be developmentally regulated and highly expressed in promastigotes. Four different leishmanial phosphodiesterases [107] have been recently cloned (PDEA, PDEB1 and PDEB2, PDEC and PDED); PDEB and PDEC are predominantly membrane bound whereas PDEA and PDED are cytosolic. These PDEs might be a controlling factor for the differentiation of the parasites as the cytosolic PDE activity decreased during stage differentiation whereas the membrane-bound PDE activity remained unaltered. Phosphodiesterase A (PDEA) belongs to the type I diesterases and the protein structurally differs in parts from its mammalian counterpart although the catalytic chains A and B are conserved. Thus inhibition by high concentrations of the mammalian inhibitors dipyramidol and triquinsin [108] can be explained but might not be useful in antiparasitic treatment. However, it remains an interesting observation that a PDEA knockout mutant decreases the peroxide concentration and triggers an antioxidant defense in the parasite. Therefore, PDEA might be an attractive target controlling the antioxidant machinery in the parasite.

## 9. Cyclic Nucleotides Activate Protein Kinases A and G without Canonical G-Proteins

The involvement of kinase cascades shows that malaria parasites are no exception in keeping these evolutionarily conserved mechanisms of signaling [109]. In *Plasmodium*, signaling can either be performed by membrane-bound adenylyl cyclase β mediating the signal over to the EPAC pathway involving the Ras GTPase Rap1 or directly to protein kinase A (Figure 1). Rap1 is directly activated by cAMP which leads to final activation of Calcium-dependent protein kinase 1 (CDPK1). In a different pathway, cAMP can directly activate protein kinase A.

The ACG group of kinases in *Plasmodium* comprises protein kinase A and G while a protein kinase C (PKC) is absent. The inactive PKA_c_ enzyme is composed of a catalytic subunit c and a regulatory subunit (PKA_r_) with cAMP binding sites. Once cAMP is bound to PKA_r_ it becomes inactive and PKA_c_ can phosphorylate its substrates. In *Plasmodium* PKA_c_ and PKA_r_ are encoded by single genes [110]. In contrast to mammalian PKA_r_, the orthologue from *Plasmodium* is missing the dimerization domain responsible for interaction with PKA activating proteins. PKA_c_ from *Plasmodium* acts at multiple stages during the parasite life cycle. In the asexual blood stages it is essential and sensitive to the inhibitors H89 (5-isoquinolinesulfonamide) and the PKI inhibitor peptide (l-threonyl-l-threonyl-l-tyrosyl-l-alanyl-l-a-aspartyl-l-phenylalanyl-l-isoleucyl-l-alanyl-l-serylglycyl-l-arginyl-l-threonylglycyl-l-arginyl-l-aspartic acid), however to a much lesser extent than the human paralogue. The role of PKA_c_ in invasion of the human host cell has been demonstrated [111]. PKA_c_ prevents increases in erythrocyte deformability [112].

Only one of the two occurring guanylyl cyclases in *Plasmodium* is important with respect to pathogenesis, i.e., PfGC_α_ which was refractory to in vivo deletion and has a vital function in the erythrocytic blood stages [87] while early studies on the function of GCβ revealed a role in ookinete motility and mosquito invasion [113]. Surprisingly, no pharmacological inhibitors have been identified yet although both proteins share structures which can only be found in the Apicomplexans i.e., C-terminal cyclase catalytic domains with purine in particular cGMP binding motifs and mammalian AC catalytic domains although they are functional guanylyl cyclases.

Among cyclic nucleotide-regulated kinases, protein kinase G (PKG) is essential in all of the key stages of the malaria parasite life cycle. The unique structural and biochemical properties i.e., three cAMP/cGMP binding motifs, a degenerated cGMP-binding motif and a lack of leucine zipper motif for dimerization make it a “druggable target”. Moreover, PKG is insensitive to cGMP analogues. In the absence of the nucleotide, a capping mechanism caused by a pseudo-substrate covers the active site. Mutations in these capping regions are lethal in the blood stages [9]. An imidazopyridine inhibitor [10] was identified which inhibited the unsual Thr_618_ gatekeeper position in *Plasmodium*. Effects of the inhibitor affected gametogenesis (no rounding up of gametocytes), ookinete gliding and blood stage development (prevention of schizont rupture). Oral dosing of a *P. falciparum* mouse model was able to clear blood-stage infection and block transmission of gametocytes to mosquitoes. A second important result is that PKG acts in concert with a stage specific calcium dependent kinase [114] (Figure 1) as in the EPAC pathway and in turn is regulating the release of calcium into the cytosol. At low potassium concentrations, an unknown ligand binds to the SR25 receptor which mediates the signal to a guanylyl cyclase that activates protein kinase G and phospholipase C and finally CDPK1. Collectively, the data show that further research on PKG in the context of drug development with respect to the clinical phase is worth being pursued.

Apart from the imidazopyrazidine inhibitor of plasmodial kinase G, a novel group of lead compounds, i.e., 3-methylisoquinoline-4 carbonitriles which inhibit protein kinase A from *Plasmodium* with promising activity in in vitro assays is now being explored [115].

Finally, apart from apparent PKA subunits in *T. cruzi* [116], no homologous genes for cAMP effectors were identified in its genome. A very peculiar feature is that the regulatory subunit of *T. brucei* PKA does not appear to bind cAMP, but instead binds cGMP [116] although there is no convincing evidence that cGMP is produced by these parasites. Thus, it is likely that PKA is not activated by cyclic nucleotides in *T. brucei*. These peculiar features might be exploited further for drug development.

Protein kinase A functions as a downstream effector in *Leishmania* parasites. Recently, two PKA catalytic subunits (PKAC) from *Leishmania* have been cloned, characterized, and found to be sensitive to mammalian PKA inhibitors [117,118] suggesting that PKA exists in *Leishmania* and perhaps plays a regulatory role in the parasite. Parasitic PKA is an inactive R_2_C_2_ heterotetramer consisting of two catalytic and two cAMP-binding regulatory subunits. Binding of cAMP to the regulatory subunits releases the active catalytic subunits, which are then free to phosphorylate a broad range of substrates. PKA is regulated by environmental signals like cAMP, pH and temperature which appears in the stage conversion of promastigotes to amastigotes. Recently the regulatory subunit from *L. donovani* has been characterized [119]. The LdPKAR protein showed extensive homology to the orthologues from *T. brucei* (60% amino acid identity) and *T. cruzi* (66% amino acid identity). Moreover, the protein is involved in cell growth and differentiation, in particular in the state where promastigotes are converted to amastigotes in autophagy (metacyclogenesis).

## 10. Conclusions

cAMP-dependent signaling pathways in the Apicomplexa and kinetoplastids are unique since canonical heterotrimeric G-proteins and GPCRs are absent in both groups. However, cAMP is essential for the stage conversion in the complex life cycles of apicomplexan parasites and kinetoplastids. Instead, membrane bound adenylyl cyclases and small GTPases may partly substitute the heterotrimeric G-proteins in signal transduction. In the Apicomplexa, mostly in *Plasmodium*, Rab families which belong to the Ras superfamily, are mainly involved in the regulation of vesicular transport and endocytic pathways during invasion of the host cell. A novel function, i.e., developmental regulation occurs in case of a not further characterized GTPase in *Cryptosporidium*. Small GTPases have not been described in *Toxoplasma gondii*, but rhoptry associated pseudokinases interact with immunity-related host GTPases Gbp2 and Irga6 during *Toxoplasma gondii* infection. However, an inhibitor has not been identified against the characterized GTPases in the Apicomplexa. In contrast, in Kinetoplastids plenty of small GTPases of the Ras family have been identified with particular functions, e.g., in endocytosis to evade the host innate immune response or flagellar motility.

Host heterotrimeric G-proteins and GPCRs are also involved in the parasite–host interaction and required for parasitic invasion. Thus additional chemotherapeutic approaches targeting canonical pathways of the human host are necessary for the eradication of the parasite. This has already been successfully shown by inhibition of the human β-adrenergic receptor with propranolol in *Plasmodium* infected human erythrocytes [23].

Secondary effectors like adenylcyclases, cAMP-dependent kinases and phosphodiesterases are present in the Apicomplexa and kinetoplastids. The most attractive target in the Apicomplexa is the small GTPase Rap-1 which is activated by cAMP through the guanine nucleotide exchange factor independently of protein kinase A. Currently, no inhibitor against Rap-1 has been identified although imidazopyrimidines inhibit calcium-dependent protein kinase1 (CDPK1) which is activated by Rap-GTP through Ca ion release in many stages of the *Plasmodium* life cycle [119].

Plenty of the secondary effectors have not only been structurally characterized but also investigated with respect to pathogenicity. Hence, the uniqueness of these proteins may be exploited in drug discovery for antiparasitic chemotherapy. Targeting of plasmodial ACG kinases has just begun. Adenosine analogue-oligoarginine conjugates inhibit a cGMP-dependent protein kinase which is a representative member of the *Plasmodium* kinome [119]. In *Toxoplasma gondii* a cAMP-dependent kinase was identified [120,121] being responsible for tachyzoite growth. The ATP-competitive inhibitor did not only have a strong effect on the protein but also inhibited tachyzoite growth. At present, there is a lack of reports on cAMP-dependent effector proteins in *Cryptosporidium*.

Adenylate cyclases, phosphodiesterases and cAMP- dependent kinases with significant differences to their human orthologues have been characterized in *Trypanosoma* and *Leishmania.* ACs are highly abundant in trypanosomes with one single transmembrane domain and dimeric function in contrast to their human counterpart. They are proposed to be mainly involved in cytokinesis and evasion of the host immune response. In particular, it remains a mystery why *T. brucei* has evolved such a high number of genome-encoded ACs [122]. To further study the link between immune invasion and resolution of plenty of incoming signals could spark an interesting process in drug development. *Leishmania major* has a soluble heme-containing AC which can be activated in response to oxygen [123]. To date, no pharmacological inhibitor against this AC in Leishmania species and the ACs in trypanosomes has been identified.

Phoshodiesterases (PDEs) have found to be essential to cell survival and virulence in the kinetoplastids. Therefore, most attempts in drug discovery focused to target these enzymes. Since many human PDEs are in clinical use one has started an unconventional approach to modify these drugs against PDEs in the pathogen.

In sum, cAMP-regulated pathways in the Apicomplexa and kinetoplastids may have a high potential for drug discovery and treatment in the future despite the absence of G-proteins and GPCRs. Impressive progress has been made in the field of secondary effectors e.g., kinases through their crystalization. However, intensive research in the future will be necessary to characterize the only sequences identified so far as “druggable targets”.
